# Pathological Phenomena Generalized

**Published:** 1861-09

**Authors:** 


ART. XIV.—*Pathological Phenomena Generalized.* By H. BACKUS. 8vo., pp. 42. Selma, Alabama.

THIS paper does not require a very extended notice at our hands; we do not feel, after perusing it, that we have added very considerably to our knowledge of the subject of pathology. The truth is, a large proportion of the pamphlet is made up of quotations from Copland, Rokitansky, Watson, and others. Congestion, as the effect of pressure, is the theme which the writer discusses on every page.

The points on which he particularly dwells are: 1. That all exciting causes of disease produce the state marked by the term congestion. 2. That this state precedes and coexists with all pathological phenomena more complex than itself. 3. That the treatment of congestion illustrates the treatment of all special diseases. 4. That this state is a constant post-mortem appearance, whether there be recognized organic change or not. The writer's object appears to be to deduce the immense variety of pathological phenomena from the state of congestion produced by pressure; and we have no doubt that he has succeeded in convincing himself of the infallibility of his reasonings.

